# A Low-Complexity Method for Two-Dimensional Direction-of-Arrival Estimation Using an L-Shaped Array

**DOI:** 10.3390/s17010190

**Published:** 2017-01-19

**Authors:** Qing Wang, Hang Yang, Hua Chen, Yangyang Dong, Laihua Wang

**Affiliations:** 1 School of Electrical and Information Engineering, Tianjin University, 92 Weijin Road, Tianjin 300072, China; wangq@tju.edu.cn (Q.W.); hang0917@tju.edu.cn (H.Y.); 2 Key Laboratory of Electronic Information Countermeasure and Simulation Technology, Ministry of Education, Xidian University, Xi’an 710071, China; dongyangyang2104@126.com; 3 School of Software, Qufu Normal University, Qufu 273165, China; wlh@tju.edu.cn

**Keywords:** low-complexity, 2D DOA estimation, L-shaped array, automatic pairing, theoretical analysis, Cramer–Rao bound

## Abstract

In this paper, a new low-complexity method for two-dimensional (2D) direction-of-arrival (DOA) estimation is proposed. Based on a cross-correlation matrix formed from the L-shaped array, the proposed algorithm obtains the automatic pairing elevation and azimuth angles without eigendecomposition, which can avoid high computational cost. In addition, the cross-correlation matrix eliminates the effect of noise, which can achieve better DOA performance. Then, the theoretical error of the algorithm is analyzed and the Cramer–Rao bound (CRB) for the direction of arrival estimation is derived . Simulation results demonstrate that, at low signal-to-noise ratios (SNRs) and with a small number of snapshots, in contrast to Tayem’s algorithm and Kikuchi’s algorithm, the proposed algorithm achieves better DOA performance with lower complexity, while, for Gu’s algorithm, the proposed algorithm has slightly inferior DOA performance but with significantly lower complexity.

## 1. Introduction

Direction-of-arrival (DOA) estimation, which has found its potential applications in the fields of sonar, radar, wireless communication, etc, is an important research branch of array signal processing [1]. Two-dimensional (2D) [2,3] direction-of-arrival (DOA) estimation with different structured arrays, such as two-parallel arrays [4,5,6,7], L-shaped arrays [8,9,10,11,12,13,14,15], and uniform rectangular array [16,17] has drawn a remarkable amount of attention. In [18], it has been proven that the estimation performance of the L-shaped array prevails over many other structured arrays. Therefore, there has been growing interest in 2D DOA estimation utilizing the L-shaped arrays. Tayem and Kwon [12] presented computationally efficient 2D angle estimation with a propagator method using one or two L-shaped arrays. Unfortunately, this method cannot pair the 2D angles automatically and may cause a matching failure problem. Consequently, a pair-matching method using a cross-correlation matrix was proposed to remove the aforementioned problem in [13]. However, the correct estimation of the incoming “virtual angles” [12] was the fatal problem at a low signal-to-noise ratio (SNRs) and with a small number of snapshots, which seriously affected the estimation performance of 2D DOAs.

A method [14] based on joint singular value decomposition (JSVD) of two cross-correlation matrices (CCMs), which mitigated the additive noise effect, was put forward to estimate elevation and azimuth parameters without additively pairing procedures. However, the method required heavy calculation due to SVD operation and “beamforming-like” spectral search operation. A two-dimensional angle matching algorithm based on the estimated signal covariance matrix is proposed in [19]. When the signal source is coherent, it can be achieved by minimizing a cost function constructed by the two covariance matrices. This method is robust to the CCM-ESPRIT algorithm. Tayem [20] divided two uniform arrays on the L-matrix into two subarrays and computed the cross-covariance matrices on the two uniform arrays, respectively. Then, adding the two mutual covariance matrices with their transpose matrix, respectively, we can obtain two new cross-covariance matrices. By segmenting these two new matrices, we can get the two-dimensional angle estimation with linear operation. However, the method still requires a two-dimensional angle matching process. By using the conjugate symmetry of two uniform linear array patterns on the L-array, the effective aperture of arrays can be extended in [21], and, then, the automatic matching of the two-dimensional angle parameters based on the PM algorithm and ESPRIT algorithm can be obtained, which avoids the cumbersome peak searching process. Therefore, the method not only has good direction finding precision, but also has the advantage of low complexity. A novel cumulants-based approach [22] to 2D DOA estimation for coherent non-Gaussian sources with two parallel ULA (uniform linear arrays) is presented. It has a lesser amount of calculation, which avoids constructing several FOC (fourth order cumulants)-based sub-matrices to form two full rank spatially smoothed matrices. When two close coherent signals are present, it is more effective and efficient than the FOC-FSS (fourth-order cumulants-based forward spatial smoothing) method in 2D DOA estimation in both white noise and color Gaussian noise situations. Wu [23] proposed a novel 2D direct-of-arrival and mutual coupling coefficients estimation algorithm for uniform rectangular arrays. The algorithm can achieve a better performance than those auxiliary sensor-based ones. It first built a general mutual coupling model that is based on banded symmetric Toeplitz matrices and then used the rank-reduction method to solve the 2D DOA estimation problem. With the obtained DOA information, the mutual coupling coefficients can be estimated.

Chen [24] derived a series of 2D DOA estimators with a new data vector that combines the received array data and its conjugate counterparts for mixed circular and non-circular signals based on a 2D array structure consisting of two parallel ULAs. However, it can give a more accurate estimation when the number of sources is within the traditional limit of high resolution methods and still work effectively when the number of mixed signals is larger than that of the array elements. In addition, it avoids the complicated 2D spectrum peak search and therefore has a much lower computational complexity. A multiresolution approach [25] for the DOA estimation of multiple signals based on a support vector classifier has been presented. This method defines a probability map of the incidence of an electromagnetic signal and performs a synthetic zoom in the angular sector iteratively. Then, it is able to estimate the DOAs of a number of sources greater than the maximum allowed by conventional eigenvalue decomposition methods for a fixed planar array geometry, and provide good results dealing with both a single signal and multiple signals.

In this paper, based on CCMs, a new pair-matching algorithm is presented to achieve 2D angles with low complexity. Firstly, the elevation angles are estimated by a linear operation of the cross-correlation matrix formed from an L-shaped array, and then the corresponding azimuth angles are achieved by the interrelationship between the elevation and azimuth angle without an additional paired procedure. Moreover, the Cramer–Rao bound (CRB) for 2D DOAs of an L-shaped array is studied. The complexity advantage of the proposed algorithm is analyzed, which is significant as sensors and snapshots increased. Furthermore, the theoretical error of the proposed algorithm is derived.

The rest of this paper is organized as follows. Section 2 presents the array signal model. Description of the proposed algorithm is introduced in Section 3. Section 4 analyzes the complexity of the proposed algorithm. The theoretical error analysis of the proposed algorithm is derived in Section 5. The analysis of the CRB of the L-shaped array is given in Section 6. The experimental results are compared with several existing approaches in Section 7. Finally, Section 8 concludes this paper.

Throughout the paper, the notations ${\left(\cdot \right)}^{*}$, ${\left(\cdot \right)}^{T}$, ${\left(\cdot \right)}^{-1}$, ${\left(\cdot \right)}^{+}$, and ${\left(\cdot \right)}^{H}$ represent conjugation, transpose, inverse, pseudo-inverse, and conjugate transpose, respectively. We use E[·] and arg(·) to separately indicate the expectation and phase angle operator.

## 2. Array Signal Model

**Figure 1:**
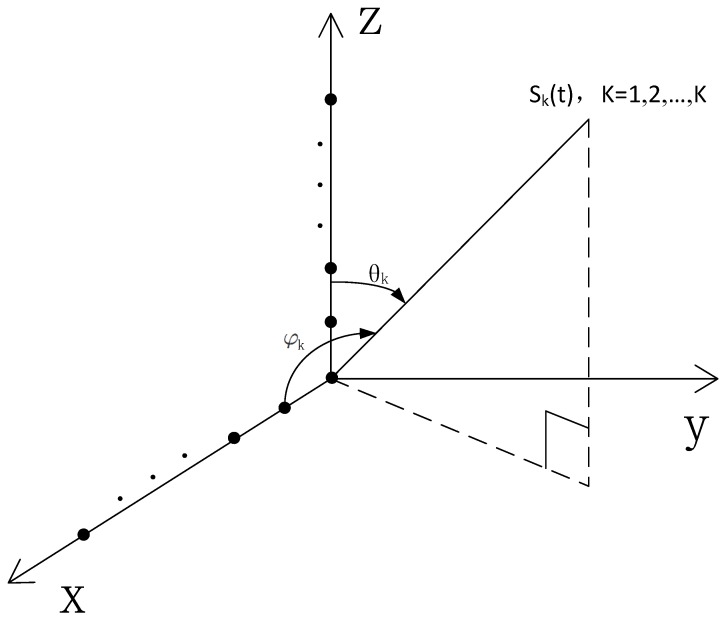
L-shaped array configuration for 2D DOA estimation.

As illustrated in Figure 1, *K* far-field narrowband plane wave signals $s_{i}\left(t\right),i=1,\ldots ,K$, impinge on the L-shaped array structured by two uniform orthogonal arrays in the *x*–*z* plane. Each array consists of *N* identical omni-directional sensors separated by *λ*/2 inter-element spacing *d*, namely, *d* = *λ*/2, where *λ* is the wavelength of the incident waves. The *i*th source has an elevation angle $\theta_{i}$ and an azimuth angle $\varphi_{i}$. The observed signal vectors at the sub-arrays along the *x*-axis and *z*-axis are written in matrix form as

(1) $$X\left(t\right)=A_{x}\left(\varphi \right)S\left(t\right)\;+\;N_{x}\left(t\right),$$

(2) $$Z\left(t\right)=A_{z}\left(\theta \right)S\left(t\right)\;+\;N_{z}\left(t\right),$$

respectively, where $X\left(t\right)={\left[x_{1}\left(t\right),x_{2}\left(t\right),\ldots ,x_{N}\left(t\right)\right]}^{T}$ and $Z\left(t\right)={\left[z_{1}\left(t\right),z_{2}\left(t\right),\ldots ,z_{N}\left(t\right)\right]}^{T}$ are the N×1 received signal vectors along the *x*-axis and *z*-axis, respectively. $S\left(t\right)={\left[s_{1}\left(t\right),s_{2}\left(t\right),\ldots ,s_{K}\left(t\right)\right]}^{T}$ is the K×1 incoming signal vector. $N_{x}\left(t\right)={\left[n_{x1}\left(t\right),n_{x2}\left(t\right),\ldots ,n_{xN}\left(t\right)\right]}^{T}$ and $N_{z}\left(t\right)={\left[n_{z1}\left(t\right),n_{z2}\left(t\right),\ldots ,n_{zN}\left(t\right)\right]}^{T}$ are the Gaussian white noise vectors along the *x*-axis and *z*-axis, respectively. In addition, $A_{x}\left(\varphi \right)=\left[a\left(\varphi_{1}\right),a\left(\varphi_{2}\right),\ldots ,a\left(\varphi_{K}\right)\right]$ and $A_{z}\left(\theta \right)=\left[a\left(\theta_{1}\right),a\left(\theta_{2}\right),\ldots ,a\left(\theta_{K}\right)\right]$ are denoted as N×K array manifold matrices of the *x*-axis and *z*-axis, respectively. N×1 array manifold vectors $a\left(\varphi_{i}\right)={\left[a_{1}\left(\varphi_{i}\right),a_{2}\left(\varphi_{i}\right),\ldots ,a_{N}\left(\varphi_{i}\right)\right]}^{T}$ and $a\left(\theta_{i}\right)={\left[a_{1}\left(\theta_{i}\right),a_{2}\left(\theta_{i}\right),\ldots ,a_{N}\left(\theta_{i}\right)\right]}^{T}$ have the form of $a_{k}\left(\varphi_{i}\right)=e^{-j(2\pi /\lambda )d_{x}\left(k-1\right)\cos\varphi_{i}}$ and $a_{k}\left(\theta_{i}\right)=e^{-j(2\pi /\lambda )d_{x}\left(k-1\right)\cos\theta_{i}}$ along the *x*-axis and *z*-axis, respectively. We suppose that the source signals are non-Gaussian and uncorrelated to each other; the Gaussian noises with zero-mean and variance $\sigma^{2}$ are statistically independent to the signals.

## 3. The Proposed Algorithm

Firstly, a cross-correlation matrix $R_{xz}$ is obtained as follows:

(3) $$\begin{array}{c} R_{xz}=E\left[X\left(t\right)Z^{H}\left(t\right)\right]\;\\ \;\;\;\;\;\;\;\;\;\;\;\;\;\;\;\;\;\;\;\;=A_{x}(\varphi )R_{s}A_{z}^{H}\left(\theta \right), \end{array}$$

where $R_{s}=E\left[S\left(t\right)S^{H}\left(t\right)\right]$. From Equation (3), it can be noted that the additive noise is removed by the cross-correlation operation. Let $R_{xz1}$ and $R_{xz2}$ be the first and last N−1 columns of $R_{xz}$, and we have

(4) $$R_{xz1}=A_{x}(\varphi )R_{s}A_{z1}^{H}\left(\theta \right),$$

(5) $$\begin{array}{c} R_{xz2}=A_{x}(\varphi )R_{s}A_{z2}^{H}\left(\theta \right)\\ \;\;\;\;\;\;\;\;\;\;\;\;\;\;\;\;\;\;\;\;\;\;\;\;=A_{x}(\varphi )R_{s}\Lambda^{H}\left(\theta \right)A_{z1}^{H}\left(\theta \right), \end{array}$$

where $A_{z2}\left(\theta \right)=A_{z1}\left(\theta \right)\Lambda \left(\theta \right)$, $\Lambda \;=\;\mathrm{diag}(e^{-j(2\pi /\lambda )d\cos\theta_{1}},\ldots ,e^{-j(2\pi /\lambda )d\cos\theta_{K}})$, $A_{z1}\left(\theta \right)$ and $A_{z2}\left(\theta \right)$ denote the first and last N−1 rows of $A_{z}\left(\theta \right)$, respectively. When the incoming signal covariance matrix $R_{s}$ is diagonal matrix, Equation (5) can be rewritten as

(6) $$R_{xz2}\;=\;A_{x}(\varphi )\Lambda^{H}R_{s}A_{z1}^{H}\left(\theta \right).$$

By combining Equations (4) and (6), a new 2N×(N−1) matrix R is defined as follows:

(7) $$\begin{array}{c} R=\left[\begin{array}{c} R_{xz1}\\ R_{xz2} \end{array}\right]\;=\;\left[\begin{array}{c} A_{x}(\varphi )\\ A_{x}(\varphi )\Lambda^{H}\left(\theta \right) \end{array}\right]R_{s}A_{z1}^{H}\left(\theta \right)\\ \;\;\;\;\;\;\;\;\;\;\;\;\;\;\;\;\;\;\;\;\;\;\;\;\;\;\;\;\;\;\;\;\;\;\;\;\;\;\;\;\;\;\;\;\;\;\;\;\;\;\;\;\;\;\;=\;A_{xe}(\varphi ,\theta )R_{s}A_{z1}^{H}\left(\theta \right). \end{array}$$

Then, we partition the matrix $A_{xe}\left(\varphi ,\theta \right)$ in Equation (7) as

(8) $$A_{xe}(\varphi ,\theta )\;=\;[A_{xe1}^{T}(\varphi ,\theta ),A_{xe2}^{T}(\varphi ,\theta {)]}^{T},$$

where $A_{xe1}\left(\varphi ,\theta \right)$ and $A_{xe2}\left(\varphi ,\theta \right)$ are the K×K and (2N−K)×K sub-matrices of $A_{xe}\left(\varphi ,\theta \right)$. Here, a K×(2N−K) propagator matrix P is defined that satisfies

(9) $$P^{H}A_{xe1}(\varphi ,\theta )\;=\;A_{xe2}(\varphi ,\theta ).$$

Similarly, we partition R in Equation (7) into K×(N−1) sub-matrix $R_{1}$ and (2N−K)×(N−1) sub-matrix $R_{2}$, which has the following relationship

(10) $$P^{H}R_{1}\;=\;R_{2}.$$

In practice, the propagator matrix P is achieved by minimizing the following cost functions

(11) $$\zeta_{csm}\left(P\right)={∥R_{2}-P^{H}R_{1}∥}_{F}^{2},$$

where ${∥\cdot ∥}_{F}^{2}$ signifies Frobenius norm. The estimate of P is as follows:

(12) $$\hat{P}={\left({\hat{R}}_{1}{\hat{R}}_{1}^{H}\right)}^{-1}{\hat{R}}_{1}{\hat{R}}_{2}^{H}.$$

To maximize usage of array information, we introduce an extended propagator matrix $P_{e}$ as follows:

(13) $$P_{e}={\left[\begin{array}{cc} I_{K}^{H} & \hat{P} \end{array}\right]}^{H},$$

where $I_{K}$ is the K×K identity matrix. In the noiseless case, right-multiplying by $A_{xe1}\left(\varphi ,\theta \right)$ of Equation (13), we obtain

(14) $$\left[\begin{array}{c} I_{K}\\ {\hat{P}}^{H} \end{array}\right]A_{xe1}(\varphi ,\theta )=A_{xe}(\varphi ,\theta )\;=\;\left[\begin{array}{c} A_{x}(\varphi )\\ A_{x}(\varphi )\Lambda^{H}\left(\theta \right) \end{array}\right].$$

Next, we partition $P_{e}$ into two N×K sub-matrices $P_{e1}$ and $P_{e2}$, and Equation (14) can be rewritten as

(15) $$\left[\begin{array}{c} P_{e1}\\ P_{e2} \end{array}\right]A_{xe1}(\varphi ,\theta )\;=\;\left[\begin{array}{c} A_{x}(\varphi )\\ A_{x}(\varphi )\Lambda^{H}\left(\theta \right) \end{array}\right].$$

According to Equation (15), we get

(16) $$P_{e1}A_{xe1}(\varphi ,\theta )\;=\;A_{x}(\varphi ),$$

(17) $$P_{e2}A_{xe1}(\varphi ,\theta )\;=\;A_{x}(\varphi )\Lambda^{H}\left(\theta \right).$$

Then, we introduce a new matrix ψ that can be expressed as

(18) $$\psi \;=\;P_{e1}^{+}P_{e2}\;=\;A_{xe1}(\varphi ,\theta )\Lambda^{H}\left(\theta \right)A_{xe1}^{-1}(\varphi ,\theta ).$$

In Equation (18), by performing eigen-value decomposition (EVD) of ψ, eigenvalues ${\hat{\alpha }}_{i}$ and corresponding eigenvectors $A_{1}^{\prime }$ that correspond to the diagonal elements of $\Lambda^{H}\left(\theta \right)$, and the estimate of $A_{xe1}(\varphi ,\theta )$ can be achieved, respectively. Here, we denote

(19) $$A_{1}^{\prime }\;=\;A_{xe1}(\varphi ,\theta )\Omega ,$$

where **Ω** is a permutation matrix with $\Omega^{-1}=\Omega$.

Then, according to the expression of $\Lambda^{H}\left(\theta \right)$, the elevation angles are as follows:

(20) $${\hat{\theta }}_{i}\;=\;\mathrm{arc}\cos\left(\arg\left({\hat{\alpha }}_{i}\right)\lambda /2\pi d\right).$$

In addition, using $P_{e11}$ to denote the first (N−1) rows of $P_{e1}$, $P_{e12}$ to denote the last (N−1) rows of $P_{e1}$, $P_{e21}$ to denote the first (N−1) rows of $P_{e2}$, and $P_{e22}$ to denote the last (N−1) rows of $P_{e2}$, respectively, we define

(21) $$B_{1}={\left[\begin{array}{cc} P_{e11}^{T} & P_{e21}^{T} \end{array}\right]}^{T}A_{1}^{\prime },$$

(22) $$B_{2}={\left[\begin{array}{cc} P_{e12}^{T} & P_{e22}^{T} \end{array}\right]}^{T}A_{1}^{\prime }.$$

With the assumption that $A^{\prime }\;=\;A_{xe}(\varphi ,\theta )\Omega$, we know that $P_{e11}A_{1}^{\prime }$, $P_{e12}A_{1}^{\prime }$, $P_{e21}A_{1}^{\prime }$, $P_{e22}A_{1}^{\prime }$ are the first (N−1) rows, the second to *N*-th row, the (N+1)-th to (2N−1)-th row, the last (N−1) rows of $A^{\prime }$, respectively, so

(23) $$P_{e11}A_{1}^{\prime }\hat{\Phi }\;=\;P_{e12}A_{1}^{\prime },$$

(24) $$P_{e21}A_{1}^{\prime }\hat{\Phi }\;=\;P_{e22}A_{1}^{\prime },$$

which contribute to

(25) $$B_{1}\hat{\Phi }\;=\;B_{2},$$

where $\hat{\Phi }\;=\;\Omega \Phi \Omega^{\;-\;1}$ with $\Phi \;=\;\mathrm{diag}(e^{-j(2\pi /\lambda )d\cos\varphi_{1}},\ldots ,e^{-j(2\pi /\lambda )d\cos\varphi_{K}})$ . In addition, the azimuth angles lie in the diagonal elements ${\hat{\beta }}_{i}$ of $B_{1}^{+}B_{2}$ as follows:

(26) $${\hat{\varphi }}_{i}\;=\;\mathrm{arc}\cos\left(-\arg\left({\hat{\beta }}_{i}\right)\lambda /2\pi d\right).$$

At this point, 2D elevation and azimuth parameters have been automatically paired by EVD operation. The summary of the proposed algorithm is shown as follows:

**Step** **1:** Compute $R_{xz}$ and R from Equations (3) and (7).
**Step** **2:** Estimate P and $P_{e}$ with Equations (12) and (13).
**Step** **3:** Execute eigen-decomposition of ψ in Equation (18).
**Step** **4:** Construct $B_{1}$ and $B_{2}$ from Equations (21) and (22).
**Step** **5:** Attain 2D elevation and azimuth from Equations (20) and (26).

## 4. Complexity Analysis

As for the complexity, we analyze on the basis of the matrix complex multiplication, which mainly involves in auto-correlation or cross-correlation matrix construction, EVD or SVD operation, pseudo-inverse operation, and “beamforming-like” spectral search. Define the search step of azimuth $\varphi \in [0,180^{\circ }]$ with $\Delta \varphi =0.01^{\circ }$. The major computations of the proposed algorithm is about $O[N^{2}L+2K^{3}+\left(7N-4\right)K^{2}+K\left(N-1\right)\left(2N-K\right)]$, while Tayem’s algorithm [12], Kikuchi’s algorithm [13], and Gu’s algorithm [14] cost approximately $O[2{\left(2N-2\right)}^{2}L+2{\left(N-1-K\right)}^{3}+2K^{3}+8\left(N-\;1\right)K^{2}+4K\left(N-1\right)\left(2N-2-K\right)+2NKL]$, $O[3N^{2}L+2N^{3}+2NKL]$, $O[N^{2}L+8N^{3}+180^{\circ }\left(N^{2}\right)/\Delta \varphi )]$, respectively, where *L* denotes the number of snapshots. Due to sample snapshots L>>N>K, therefore, the proposed algorithm has lower complexity than others.

**Figure 2:**
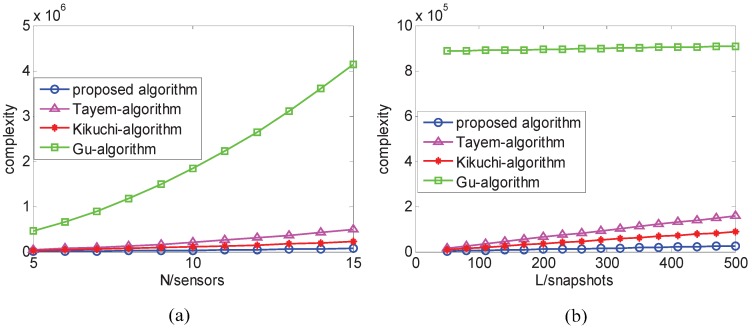
(**a**) Complexity comparison versus sensors; and (**b**) complexity comparison versus snapshots.

Figure 2a,b shows the complexity comparison between the proposed method and other methods. From both Figure 2a,b, we find that the proposed method has lower computational load than others as sensors and snapshots increase.

## 5. Theoretical Performance Analysis

The perturbation is caused by noise in the proposed method, and we analyze on the basis of the matrix perturbation theory [26,27].

Let $\hat{X}\;=\;X\;+\;\Delta X,\;\hat{Z}=Z+\Delta Z$, and the covariance matrix with perturbation be expressed as

(27) $$\begin{array}{c} {\hat{R}}_{xz}=R_{xz}+\Delta R_{xz}\\ \;\;\;\;\;\;=E\left(XZ^{H}\right)+E\left(X\Delta Z^{H}\right)+E\left(\Delta XZ^{H}\right)+E\left(\Delta X\Delta Z^{H}\right), \end{array}$$

where $\Delta R_{xz}$ is the perturbation of the covariance matrix.

Then, ${\hat{R}}_{xz1}=R_{xz1}+\Delta R_{xz1}$, ${\hat{R}}_{xz2}=R_{xz2}\;+\;\Delta R_{xz2}$ where $\Delta R_{xz1},\Delta R_{xz2}$ is the first and last N−1 columns of $\Delta R_{xz}$ . From Equation (7), we can get

(28) $$\begin{array}{c} \hat{R}=R+\Delta R\\ \;\;\;\;=\left[\begin{array}{c} R_{xz1}\\ R_{xz2} \end{array}\right]+\left[\begin{array}{c} \Delta R_{xz1}\\ \Delta R_{xz2} \end{array}\right]. \end{array}$$

${\hat{R}}_{1}=R_{1}+\Delta R_{1},\;{\hat{R}}_{2}=R_{2}+\Delta R_{2}$, where $\Delta R_{1},\Delta R_{2}$ are the first *K* rows and the last (2N−1) rows of ΔR, respectively.

From Equation (10), we get $\;{\left(P+\Delta P\right)}^{H}\left(R_{1}+\Delta R_{1}\right)=R_{2}+\Delta R_{2}$, according to $P^{H}R_{1}=R_{2}$ and, neglecting the second-order term $\Delta P^{H}\Delta R_{1}$, we can get $\Delta P^{H}$

(29) $$\Delta P^{H}=\left(\Delta R_{2}-P^{H}\Delta R_{1}\right)R_{1}^{+}.$$

The extended propagator matrix ${\hat{P}}_{e}$ is as follows:

(30) $$\begin{array}{c} {\hat{P}}_{e}=P_{e}+\Delta P_{e}\\ \;\;\;\;\;={\left[I_{K}^{H}\;P\right]}^{H}+{\left[0_{K}^{H}\;\Delta P\right]}^{H} \end{array}$$

and ${\hat{P}}_{e1}=P_{e1}+\Delta P_{e1},\;{\hat{P}}_{e2}=P_{e2}+\Delta P_{e2}$, where $\Delta P_{e1},\;\Delta P_{e2}$ are the first and last *N* rows of ΔP. According to Equation (18), $\hat{\psi }=\;\psi +\Delta \psi ,\;\psi =P_{e1}^{+}P_{e2}$ . Similar to Equation (29), we can get $\Delta \psi =P_{e1}^{+}\left(\Delta P_{e2}-\Delta P_{e1}\psi \right)$.

By performing EVD of $\hat{\psi }$ with perturbation, the influence to eigenvalues $\alpha_{i}$ can be expressed as ${\hat{\alpha }}_{i}=\alpha_{i}+\Delta \alpha_{i}$ and $\;\Delta \alpha_{i}=v_{i}\Delta \psi_{i}u_{i}$, where $v_{i}$ and $u_{i}$ stand for the left and right orthogonal eigenvectors associated with $\alpha_{i}$ of ψ, respectively.

Let $\phi_{i}=\arg\left(\alpha_{i}\right)$. Then, Equation (20) can be written as $\theta_{i}=\arccos\left(\phi_{i}\lambda /2\pi d\right),\;{\hat{\theta }}_{i}\;=\;\theta_{i}\;+\;\Delta \theta_{i}$. The perturbations of elevation $\Delta \theta_{i}$ can be obtained according to the theorem of first-order approximation of multivariate function [28]. Specific content is as follows.

For *z* close to *x*, the first-order approximation of *f* near *x* can be represented as:

(31) $$f\left(z\right)=f\left(x\right)+\nabla f{\left(x\right)}^{T}\left(z-x\right),$$

where ∇f(x) denotes the gradient of *f* and is a column vector. Thus, we can get

(32) Δθi=∂θi∂ϕi×Δϕi=Dθ×Δϕi=Dθ×Im(Δαiαi),

where $D_{\theta }=-\frac{\lambda /2\pi d}{\sqrt{1-{\left(\frac{\arg(\alpha_{i})\lambda }{2\pi d}\right)}^{2}}}$.

For Equations (21) and (22), the perturbations are

(33) $${\hat{B}}_{1}=\left[\begin{array}{c} {\hat{P}}_{e11}\\ {\hat{P}}_{e21} \end{array}\right]\hat{{A_{1}}^{\prime }}=\left[\begin{array}{c} P_{e11}\\ P_{e21} \end{array}\right]{A_{1}}^{\prime }+\left[\begin{array}{c} \Delta P_{e11}\\ \Delta P_{e21} \end{array}\right]{A_{1}}^{\prime }+\left[\begin{array}{c} P_{e11}\\ P_{e21} \end{array}\right]\Delta {A_{1}}^{\prime }+\left[\begin{array}{c} \Delta P_{e11}\\ \Delta P_{e21} \end{array}\right]\Delta {A_{1}}^{\prime }$$

(34) $${\hat{B}}_{2}=\left[\begin{array}{c} {\hat{P}}_{e12}\\ {\hat{P}}_{e22} \end{array}\right]\hat{{A_{1}}^{\prime }}=\left[\begin{array}{c} P_{e12}\\ P_{e22} \end{array}\right]{A_{1}}^{\prime }+\left[\begin{array}{c} \Delta P_{e12}\\ \Delta P_{e22} \end{array}\right]{A_{1}}^{\prime }+\left[\begin{array}{c} P_{e12}\\ P_{e22} \end{array}\right]\Delta {A_{1}}^{\prime }+\left[\begin{array}{c} \Delta P_{e12}\\ \Delta P_{e22} \end{array}\right]\Delta {A_{1}}^{\prime }$$

and $\hat{{A_{1}}^{\prime }}={A_{1}}^{\prime }+\Delta {A_{1}}^{\prime }$, where $\Delta {A_{1}}^{\prime }$ is the estimation error of ${A_{1}}^{\prime }$.

According to Equation (25), $\hat{\Phi }=\Phi +\Delta \Phi$, and $\Delta \Phi =B_{1}^{+}\left(\Delta B_{2}-\Delta B_{1}\Phi \right)$ can be obtained with a similar method to Equation (29). It can be easily obtained that the perturbations of diagonal elements $\beta_{i}$ are the diagonal elements of ΔΦ. Let $\zeta_{i}=\arg\left(\beta_{i}\right)$, and the perturbations of the azimuth can be expressed as follows, similar to elevation:

(35) Δφi=∂φi∂ζi×Δζi=Dφ×Im(Δβiβi),

where $D_{\varphi }=\frac{\lambda /2\pi d}{\sqrt{1-{\left(\frac{\arg(\beta_{i})\lambda }{2\pi d}\right)}^{2}}}$.

Therefore, the root mean-squared error of two-dimensional direction of arrival estimations are

(36) $$\Delta \theta_{i}=D_{\theta }\cdot Im\left(\frac{\Delta \alpha_{i}}{\alpha_{i}}\right),$$

(37) $$\Delta \varphi_{i}=D_{\varphi }^{}\cdot Im\left(\frac{\Delta \beta_{i}}{\beta_{i}}\right).$$

## 6. Cramer–Rao Bound (CRB) Analysis

In the case of L-shaped array configuration, the Cramer–Rao bound (CRB) of 2D DOAs is considered here. Rewrite the received data from L-shaped array as

(38) $$\begin{array}{c} Y\left(t\right)=\left[\begin{array}{c} X(t)\\ Z(t) \end{array}\right]=\left[\begin{array}{c} A_{x}\left(\varphi \right)\\ A_{z}\left(\theta \right) \end{array}\right]S\left(t\right)\;+\;\left[\begin{array}{c} N_{x}\left(t\right)\\ N_{z}\left(t\right) \end{array}\right]\\ \;\;\;\;\;\;\;\;\;\;\;\;\;\;\;\;\;\;\;\;\;\;\;\;\;\;\;\;\;\;\;\;\;\;\;\;=\;AS(t)\;+\;N. \end{array}$$

The Fisher information matrix (FIM) **F** with respect to $\varphi =[\varphi_{1},\varphi_{2},\ldots ,\varphi_{K}]$ and $\theta =[\theta_{1},\theta_{2},\ldots ,\theta_{K}]$ can be written as

(39) $$F=\left[\begin{array}{cc} F_{11} & F_{12}\\ F_{21} & F_{22} \end{array}\right].$$

Note that the (i,j)-th element of $F_{11}$ [29] is given by

(40) F(φi,φj)=2Re{trace[(A˙φiS)Hγ−1(A˙φjS)]}=2Re{trace[(A˙φeieiTS)Hγ−1(A˙φejejTS)]}=2Re[(eiTA˙φHγ−1A˙φej)(ejTSSHei)]=2LRe[(A˙φHγ−1A˙φ)ij(RsT)ij].

Similarly, we get the (i,j)-th element of $F_{12}$, $F_{21}$ and $F_{22}$, respectively, as follows:

(41) F(φi,θj)=2Re{trace[(A˙φiS)Hγ−1(A˙θjS)]}=2Re{trace[(A˙φeieiTS)Hγ−1(A˙θejejTS)]}=2Re[(eiTA˙φHγ−1A˙θej)(ejTSSHei)]=2LRe[(A˙φHγ−1A˙θ)ij(RsT)ij],

(42) F(θi,φj)=2Re{trace[(A˙θiS)Hγ−1(A˙φjS)]}=2Re{trace[(A˙θeieiTS)Hγ−1(A˙φejejTS)]}=2Re[(eiTA˙θHγ−1A˙φej)(ejTSSHei)]=2LRe[(A˙θHγ−1A˙φ)ij(RsT)ij],

(43) F(θi,θj)=2Re{trace[(A˙θiS)Hγ−1(A˙θjS)]}=2Re{trace[(A˙θeieiTS)Hγ−1(A˙θejejTS)]}=2Re[(eiTA˙θHγ−1A˙θej)(ejTSSHei)]=2LRe[(A˙θHγ−1A˙θ)ij(RsT)ij],

where Re(·) denotes the real part, $e_{i}$ denotes the *i*-th column of the identity matrix, trace(·) denotes the trace of a matrix and $M_{ij}$ denotes the (i,j)-th element of M, ${\dot{A}}_{\varsigma_{m}}$, ${\dot{A}}_{\varsigma }$(m=i,j,ς=φ,θ), $R_{s}$ and *γ* has the form of

(44) $${\dot{A}}_{\varsigma_{m}}\;=\;\frac{\partial A}{\partial \varsigma_{m}},$$

(45) $${\dot{A}}_{\varsigma }\;=\;\left[\begin{array}{c} \frac{\partial A}{\partial \varsigma_{1}},\frac{\partial A}{\partial \varsigma_{2}},...,\frac{\partial A}{\partial \varsigma_{K}}\\ 0_{N\times K} \end{array}\right],$$

(46) $$R_{s}\;=\;\frac{1}{L}SS^{H},$$

(47) $$\gamma \;=\;\left[\begin{array}{cc} Q & 0\\ 0 & Q \end{array}\right].$$

In Equation (47), Q has different expressions for different type of noises as below:

(48) $$Q=\left\{\begin{array}{cc} I_{N}, & \mathrm{for}\;\mathrm{white}\;\mathrm{noise},\\ P, & \mathrm{for}\;\mathrm{unknown}\;\mathrm{noise}, \end{array}\right.$$

where $I_{N}$ denotes the N×N identify matrix, and the (p,q)-th element of the unknown noise covariance matrix P is $0.8^{\left|p-q\right|}e^{\frac{j(p-q)\pi }{2}}.$

According to Equations (40) to (43), we obtain

(49) F11=2LRe[(A˙φHγ−1A˙φ)⊗(RsT)],

(50) F12=2LRe[(A˙φHγ−1A˙θ)⊗(RsT)],

(51) F21=2LRe[(A˙θHγ−1A˙φ)⊗(RsT)],

(52) F22=2LRe[(A˙θHγ−1A˙θ)⊗(RsT)],

where ⊗ denotes the Hadamard matrix product.

Then, the CRB matrix C can be expressed as

(53) $$C=F^{-1},$$

and we can obtain the CRB of azimuth and elevation parameters as follows:

(54) $${\mathrm{CRB}}_{\varphi_{i}}=C_{i,i}\;\;\;,$$

(55) $${\mathrm{CRB}}_{\theta_{i}}=C_{i+K,i+K}\;,$$

where $C_{i,i}$ denotes the (i,i)-th element of C.

Therefore, we define the CRB for the parameters of the *i*-th source as

(56) $${\mathrm{CRB}}_{i}=\sqrt{C_{i,i}+C_{i+K,i+K}}\;\;\;\;\;\;\;\;\;\;\;\;\;\;\;\;\;i=1,2,...,K.$$

## 7. Experimental Results

Simulation experiments are conducted in this part. In all experiments, the elements spacing of L-shaped array is *λ*/2.

In the first experiment, we examine the scattergram of 2D elevation and azimuth of the proposed algorithm compared with that of the Kikuchi algorithm in both white noise and unknown noise situations. The number of isotropic sensors *N* is 5. Two uncorrelated equal power signals with elevation $\theta_{i}$ and azimuth $\varphi_{i}$ incoming separately from $\left(55^{\circ },65^{\circ }\right)$ and $\left(75^{\circ },80^{\circ }\right)$. In addition, their SNRs are set to 20 dB and the number of snapshots are fixed at 300. Five hundred independent trials are carried out.

**Figure 3:**
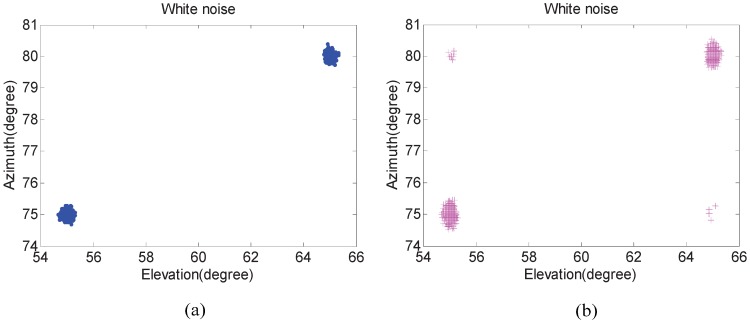
The angle estimation scattergram in a white noise situation. (**a**) The proposed algorithm; and (**b**) the Kikuchi algorithm.

**Figure 4:**
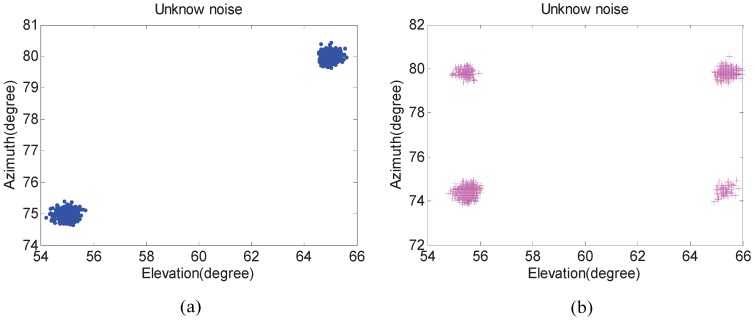
The angle estimation scattergram in an unknown noise situation. (**a**) The proposed algorithm; and (**b**) the Kikuchi algorithm.

Figure 3 and Figure 4 show that 2D DOA statistic performance of the proposed algorithm is better than the Kikuchi algorithm, especially in an unknown noise situation. In addition, pairing failures are emerging in Figure 3b and Figure 4b. The reason is that the noise factor in the proposed algorithm has been removed, and the difference between “virtual angles” is small in the Kikuchi algorithm when pair-matching is required.

In the second experiment, the proposed algorithm in theoretical analysis and experimental studies, Tayem’s algorithm, Kikuchi’s algorithm, Gu’s algorithm and CRB are compared in terms of root mean square error (RMSE) with respect to SNRs and snapshots in white noise situations. Define RMSE as

(57) $${\mathrm{RMSE}}_{i}=\sqrt{\frac{1}{1000}\sum_{l=1}^{1000}[{\left({\hat{\varphi }}_{i,l}-\varphi_{i}\right)}^{2}\;+\;{\left({\hat{\theta }}_{i,l}-\theta_{i}\right)}^{2}]}\;\;\;\;\;\;\;\;\;i=1,2,...,K.$$

**Figure 5:**
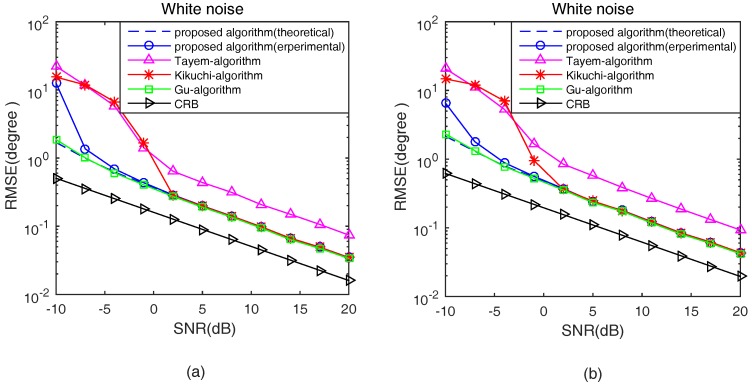
RMSE versus SNRs in a white noise situation. (**a**) $s_{1}\left(t\right)$; and (**b**) $s_{2}\left(t\right)$.

**Figure 6:**
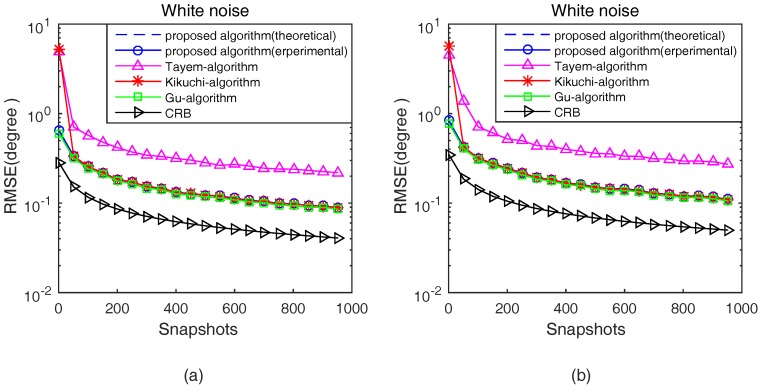
RMSE versus Snapshots in a white noise situation.(**a**) $s_{1}\left(t\right)$; and (**b**) $s_{2}\left(t\right)$.

The number of isotropic sensors *N* is 7. The 2D angle parameters of two signals with equal power are from the incident direction $\left[\varphi_{1},\theta_{1}\right]=\left[80^{\circ },65^{\circ }\right]$, $\left[\varphi_{2},\theta_{2}\right]=\left[55^{\circ },45^{\circ }\right]$. Figure 5 and Figure 6 show the 2D angle estimation performance with 200 sampling snapshots and 5dB, respectively, in a white noise situation. In addition, 1000 Monte Carlo trials are conducted in Figure 4 and Figure 5.

From Figure 5 and Figure 6, it can be noted that the theoretical estimation performance of the proposed algorithm is better than the experimental at low SNR, and, with the increase of SNR and snapshots, they gradually overlap together. In addition, the proposed algorithm is better than Tayem’s algorithm, Kikuchi’s algorithm, but slightly inferior to Gu’s algorithm at low SNR and with a small number of snapshots. As the SNR and snapshots increased, the estimation performance of the proposed algorithm is close to Gu’s algorithm with lower computational cost, which avoids SVD of the cross-correlation matrix R and “beamforming-like” spectral search.

**Figure 7:**
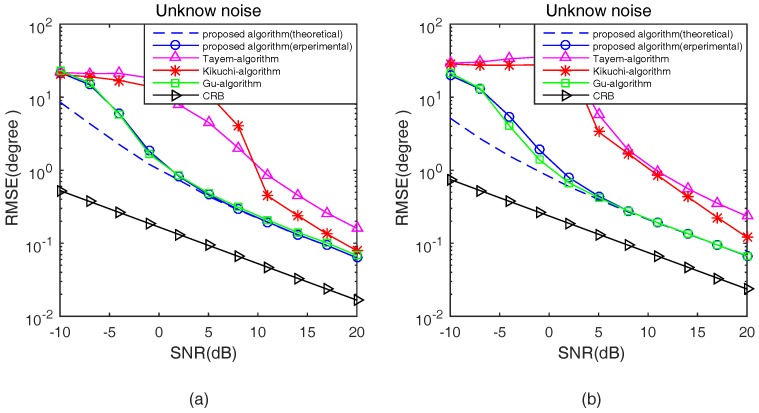
RMSE versus SNR in an unknown noise situation. (**a**) $s_{1}\left(t\right)$; and (**b**) $s_{2}\left(t\right)$.

**Figure 8:**
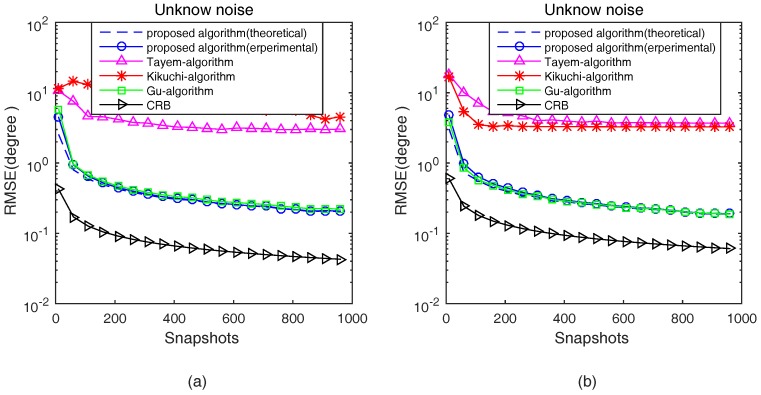
RMSE versus Snapshots in an unknown noise situation.(**a**) $s_{1}\left(t\right)$; and (**b**) $s_{2}\left(t\right)$.

In the third experiment, the proposed algorithm in theoretical analysis and experimental studies, Tayem’s algorithm, Kikuchi’s algorithm, Gu’s algorithm and CRB are compared in terms of RMSE with respect to SNRs and snapshots in an unknown noise situation. The parameters configured in this experiment are the same as the second experiment. Figure 7 and Figure 8 show the 2D DOA statistic performance in an unknown noise situation.

Apparently, as shown in Figure 7 and Figure 8, similar conclusions can be drawn. From Figure 7 and Figure 8, it can be noted that the trend of theoretical and experimental estimation performance of the proposed algorithm is the same as Figure 5 and Figure 6. Then, we can get that the DOA estimation performance of Tayem’s algorithm and Kikuchi’s algorithm deteriorates seriously because Tayem’s algorithm and Kikuchi’s algorithm are sensitive to the type of noise. In addition, the estimation performance of the proposed algorithm is roughly the same as Gu’s algorithm at low SNR and with a small number of snapshots. At high SNR and with a large number of snapshots, the estimation performance of the proposed algorithm is very close to Gu’s algorithm with lower computational cost.

## 8. Conclusions

A novel low-complexity method for 2D angle parameter estimation is proposed in this paper. The explicit description of the proposed method is derived to achieve the automatic pairing 2D angle parameters. In addition, the theoretical performance analysis and CRB of 2D DOAs is given. Simulation results show the effectiveness of the proposed algorithm in contrast to other algorithms, especially at low SNR and with a small number of snapshots.

## Abbreviations

The following abbreviations are used in this manuscript:

DOA	Direction-of-Arrival
CRB	Cramer–Rao Bound
SNR	Signal-to-Noise Ratio
JSVD	Joint Singular Value Decomposition
CCMs	Cross-Correlation Matrices
